# Directly and Indirectly Targeting the Glycine Modulatory Site to Modulate NMDA Receptor Function to Address Unmet Medical Needs of Patients With Schizophrenia

**DOI:** 10.3389/fpsyt.2021.742058

**Published:** 2021-10-01

**Authors:** Ju-Chun Pei, Da-Zhong Luo, Shiang-Shin Gau, Chia-Yuan Chang, Wen-Sung Lai

**Affiliations:** ^1^Department of Psychology, National Taiwan University, Taipei, Taiwan; ^2^Neurobiology and Cognitive Science Center, National Taiwan University, Taipei, Taiwan; ^3^Graduate Institute of Brain and Mind Sciences, National Taiwan University, Taipei, Taiwan

**Keywords:** schizophrenia, unmet medical need, negative symptoms, cognitive impairments, glycine modulatory site (GMS), d-serine, glycine transporter 1 (GlyT1) inhibitor, D-amino acid oxidase (DAO) inhibitor

## Abstract

Schizophrenia is a severe mental illness that affects ~1% of the world's population. It is clinically characterized by positive, negative, and cognitive symptoms. Currently available antipsychotic medications are relatively ineffective in improving negative and cognitive deficits, which are related to a patient's functional outcomes and quality of life. Negative symptoms and cognitive deficits are unmet by the antipsychotic medications developed to date. In recent decades, compelling animal and clinical studies have supported the NMDA receptor (NMDAR) hypofunction hypothesis of schizophrenia and have suggested some promising therapeutic agents. Notably, several NMDAR-enhancing agents, especially those that function through the glycine modulatory site (GMS) of NMDAR, cause significant reduction in psychotic and cognitive symptoms in patients with schizophrenia. Given that the NMDAR-mediated signaling pathway has been implicated in cognitive/social functions and that GMS is a potential therapeutic target for enhancing the activation of NMDARs, there is great interest in investigating the effects of direct and indirect GMS modulators and their therapeutic potential. In this review, we focus on describing preclinical and clinical studies of direct and indirect GMS modulators in the treatment of schizophrenia, including glycine, D-cycloserine, D-serine, glycine transporter 1 (GlyT1) inhibitors, and D-amino acid oxidase (DAO or DAAO) inhibitors. We highlight some of the most promising recently developed pharmacological compounds designed to either directly or indirectly target GMS and thus augment NMDAR function to treat the cognitive and negative symptoms of schizophrenia. Overall, the current findings suggest that indirectly targeting of GMS appears to be more beneficial and leads to less adverse effects than direct targeting of GMS to modulate NMDAR functions. Indirect GMS modulators, especially GlyT1 inhibitors and DAO inhibitors, open new avenues for the treatment of unmet medical needs for patients with schizophrenia.

## Introduction to Schizophrenia and Unmet Medical Needs in Patient With Schizophrenia

Schizophrenia is a devastating mental illness, and the lifetime prevalence of schizophrenia is ~1%. Globally, there were 1.13 million schizophrenia cases and 12.66 million DALYs (disability-adjusted life years) due to schizophrenia in 2017 (1). The global burden of schizophrenia remains large and continues to increase, increasing the burden on health-care systems worldwide. This debilitating brain disorder typically emerges in late adolescence and early adulthood and is characterized by three main symptoms: positive symptoms, negative symptoms, and cognitive deficits (2, 3). Positive symptoms include delusions, hallucinations, and disorganized thoughts and speech typically regarded as manifestations of psychosis. Negative symptoms include reduced affect display, alogia, anhedonia, asociality, avolition, lack of emotional response, and motivation. Cognitive deficits include dysfunctions in working memory, attention, processing speed, visual and verbal learning with substantial deficits in reasoning, planning, abstract thinking, and problem solving. Cognitive impairments and negative symptoms, as the core features of schizophrenia, are enduring and correlate with the degree of disability (4, 5).

Currently, antipsychotic medications are mainstays in the treatment of schizophrenia and a range of other psychotic disorders. Positive symptoms of schizophrenia often respond well to antipsychotic drugs. In contrast, the available antipsychotic medications, which mainly affect the dopamine and serotonin receptor systems, are relatively ineffective in improving negative and cognitive deficits. Negative symptoms of schizophrenia tend to linger or worsen over time and are accompanied by impaired cognitive function in patients with schizophrenia (6). The improvement of cognitive dysfunction is a better predictor of patient quality of life (7, 8). Since existing pharmacological and biological therapeutic modalities fail to improve cognitive symptoms, various cognitive remediation strategies have been adopted (9). In addition, the cognitive deficits in adolescents at risk for schizophrenia and in patients after their first episode of schizophrenia suggest that schizophrenia-related cognitive dysfunction is not the result of chronic illness (10). The US National Institute of Mental Health (NIMH) thus developed the Measurement and Treatment Research to Improve Cognition in Schizophrenia (MATRICS), which significantly raised awareness of the cognitive dysfunction in schizophrenia (11). In addition to the reliance on the dopamine receptor D2 (DRD2) as a conventional therapeutic target (12), a focus on the different symptom domains of schizophrenia may lead to the identification of different endophenotypic markers that can promote the development of novel therapeutics useful for rational cellular and molecular targets.

## The Roles of Glutamatergic Transmission and NMDAR (N-Methyl-D-Aspartate Receptor) Hypofunction in the Pathophysiology of Schizophrenia

Similar to those of many other psychiatric disorders, the etiology and pathophysiology of schizophrenia remain unclear. Accumulating evidence from human genetic studies and association studies has revealed several schizophrenia susceptibility loci and genes. A genome-wide association study (GWAS) revealed notable associations relevant to the major hypotheses of the etiology and treatment of schizophrenia, including *DRD2* (the main target of many effective antipsychotics) and multiple genes [e.g., *metabotropic glutamate receptor 3 (GRM3*), *glutamate ionotropic receptor NMDA type subunit 2A (GRIN2A*), *serine racemase (SR)*, and *glutamate receptor, ionotropic, AMPA receptor 1 (GRIA1*)] involved in glutamatergic neurotransmission and synaptic plasticity (13). In contrast to the conventional view of dopamine involvement in schizophrenia (i.e., the dopamine hypothesis of schizophrenia), glutamatergic neurotransmission has been gradually attracting attention in the investigation of the pathophysiology and treatment of schizophrenia in recent decades (14–16).

**Figure 1:**
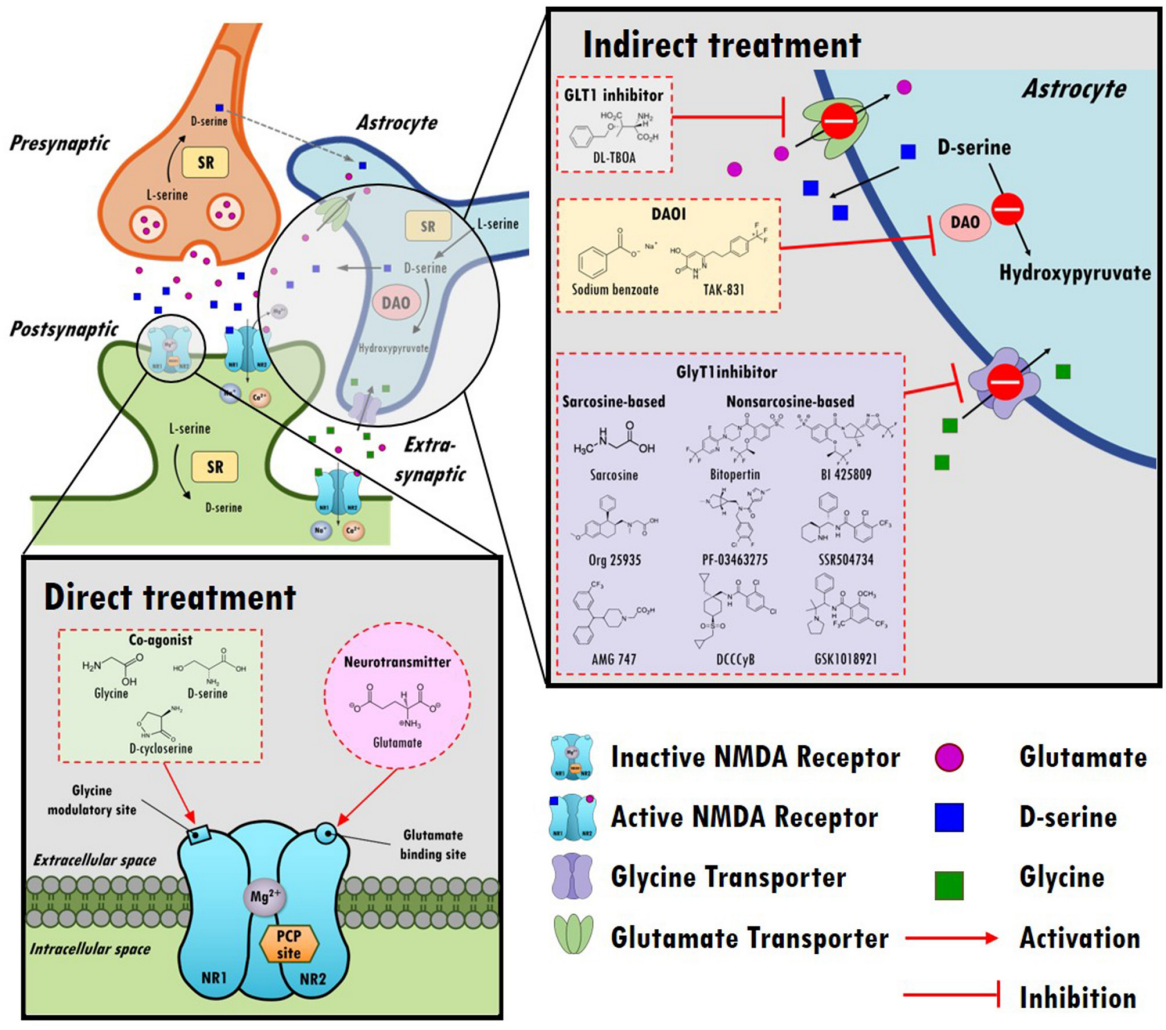
An overview of the hypothesis of N-methyl-D-aspartate receptor (NMDAR) hypofunction in schizophrenia and the direct/indirect treatments in the regulation of NMDAR functioning. Top left panel: A model of glutamatergic trisynapses: pre-synapses, post-synapses, and astrocytes. Activation of NMDAR requires not only the binding of glutamate to the GluN2 (NR2) subunit but also the binding of the coagonist glycine or D-serine at the glycine modulatory site (GMS) of the GluN1 (NR1) subunit. In response to NMDAR activation, the intracellular calcium concentration increases and thereby triggers downstream signaling cascades. After activation, glutamate and glycine are taken up by astrocytes through the glutamate transporter (GLT1) and glycine transporter (GlyT1), respectively. D-serine, another coagonist of GMS, is predominantly produced in neurons, is synthesized from L-serine by serine racemase (SR) and is shuttled to astrocytes, where it is stored and released. NMDARs are critical for synaptic plasticity, cortical maturation, and learning and memory processes. The hypofunction of ionotropic glutamate NMDARs has been proposed to be a model of schizophrenia in humans, and NMDAR hypofunction plays a key role in the pathophysiology of schizophrenia. Bottom left panel: Enhancing NMDAR functions through direct treatments. Glutamate, glycine, D-cycloserine, and D-serine compounds directly target postsynaptic NMDAR and activate NMDAR functioning. Top right panel: Boosting NMDAR functions via indirect treatments (e.g., GLT1 inhibitors, DAO inhibitors, and GlyT1 inhibitors). GLT1 inhibitors block the reuptake of glutamate and increase the synaptic levels of glutamate. D-serine is metabolized into hydroxypyruvate by D-amino acid oxidase (DAO) in astrocytes. DAO inhibitors block the metabolism of D-serine, which prolongs the synaptic concentration of D-serine. GlyT1 inhibitors block the reuptake of glycine and increase synaptic levels of glycine.

In the central nervous system (CNS), glutamate is the main excitatory neurotransmitter and activates metabotropic and ionotropic glutamate receptors. NMDARs are ionotropic glutamate-gated cation channels with high calcium permeability that play vital roles in synaptic transmission, neuroplasticity, and cognitive functions. Heterotetrameric NMDARs are widely distributed throughout most of the brain and are composed of two obligatory GluN1 (NR1) subunits with either two GluN2 (NR2) subunits or a combination of GluN2 (NR2) and GluN3 (NR3) subunits. As illustrated in the top left panel of Figure 1, activation of NMDARs requires not only the binding of glutamate on the GluN2 subunit but also the binding of the coagonist glycine or D-serine at the glycine modulatory site (GMS, also referred to as the glycine-B site or the strychnine-insensitive glycine site) on the GluN1 subunit (17). Intriguingly, although the endogenous high-potency coagonists glycine and D-serine are present in the extracellular space (18), the GMSs on NMDARs are not saturated *in vivo* (19). D-serine appears to be the dominant endogenous coagonist for NMDARs and a modulator for NMDAR-related neurotoxicity, even though the levels of glycine are 10-fold higher than those of D-serine (20–22). The activation of NMDARs produces prolonged increases in intracellular calcium concentration and thus triggers downstream signaling cascades involved in the regulation of many physiological and pathophysiological processes (23).

NMDAR has been proposed to be an important and potential therapeutic target for many CNS and psychiatric disorders (24). There is increasing evidence acquired through different approaches supports the supposition that NMDAR hypofunction plays a role in schizophrenia. In addition to the abovementioned large-scale GWAS, copy number variant studies have also led to the identification of rare genetic variants in NMDAR-related genes and components related to the postsynaptic density associated with increased risk for schizophrenia (25, 26). Postmortem brain studies have also indicated decreased expression of the NR1 subunit (mRNA and protein) and NR2C subunit (mRNA) in the postmortem dorsolateral prefrontal cortex in schizophrenic patients (27) and reductions in D-serine and serine racemase (SR) levels in patients with schizophrenia (28). A meta-analysis study further indicated significant decreases in the expression of NR1 mRNA and protein in the prefrontal cortex of schizophrenic patients (29). In addition to these genetic and postmortem studies, aberrant NMDAR function has been identified via the use of psychotomimetic agents. Pharmacological studies have revealed that the use of NMDAR antagonists (e.g., phencyclidine (PCP) and ketamine) causes not only positive symptoms of schizophrenia but also negative symptoms and cognitive deficits in healthy humans (30–32). Subanesthetic doses of ketamine not only induce psychotomimetic effects but also increase amphetamine-induced dopamine release in the striatum, which has been observed in schizophrenic patients (33). In addition, positron emission tomography (PET) imaging data have indicated links between glutamatergic system dysfunction and schizophrenia (34). NMDAR hypofunction in parvalbumin (PV) interneurons has also been proposed as a pathological mechanism of schizophrenia (35). Proton magnetic resonance spectroscopy (MRS) studies have revealed increased glutamine levels in the medial prefrontal cortex, anterior cingulate cortex, and thalamus in drug-naïve patients with first-episode psychosis (36, 37), suggesting dysregulation of glutamate neurotransmission (38). Moreover, reduced activation of the prefrontal cortices (i.e., hypofrontality) has been considered to underlie negative symptoms and cognitive deficits in schizophrenia (39–41). Notably, it has been proposed that antipsychotic medications may reduce NMDARs activity and produce dysfunctions in the corticolimbothalamic circuit and hypofrontality in patients with schizophrenia (42). Accordingly, these studies indicate the involvement of NMDARs in the pathophysiology of schizophrenia and provide new potential targets for the treatment of schizophrenia.

Given the importance of glutamate in the NMDAR hypofunction hypothesis for schizophrenia and NMDAR-mediated neurotransmission, one possible strategy to boost NMDAR functions involves either directly or indirectly enhancing glutamate levels in synapses, as illustrated in the bottom left and top right portions of Figure 1. However, excessive glutamate induces high levels of calcium influx, which has been shown to lead to excitotoxicity and neuronal injury in cellular and animal models (43, 44). In addition, indirect enhancement of glutamate via DL-TBOA, a glutamate transporter 1 (GLT1) inhibitor, resulted in attenuated baroreflex control of sympathetic nerve activity and heart rate (45). Apparently, from a safety perspective, neither direct nor indirect enhancement of synaptic glutamate levels is a reasonable therapeutic approach in the regulation of NMDAR functions. Alternatively, agents that act at the GMSs of NMDARs have been proposed to be promising treatments to moderate severe negative symptoms and cognitive impairments.

## Directly Targeting the GMS on NMDARs

A unique characteristic of NMDAR is that the GMS must be occupied by glycine and/or D-serine for glutamate to induce channel opening. GMS was first reported by Johnson and Ascher to facilitate the activation of NMDARs in cultured mouse brain neurons (18). It was later demonstrated that glycine is necessary to activate NMDARs (46). Mice carrying targeted point mutations in the GMS of the NMDAR NR1 subunit gene (*Grin1*) exhibited marked NMDAR hypofunctions and deficits in long-term potentiation and spatial learning (47, 48), as well as impaired social ability and spatial recognition (49). Accumulating evidence has indicated that binding to the GMS can enhance the affinity and efficacy of glutamate neurotransmission (50), and the administration of GMS agonists (e.g., glycine) can benefit schizophrenic patients by regulating NMDAR-mediated neurotransmission (19). The disturbance of GMS modulators found in schizophrenia patients has been identified as a contributor to NMDAR hypofunction. Previous studies have revealed reduced D-serine and SR in schizophrenia (28). In addition, the levels of kynurenic acid, the only known competitively endogenous antagonist of the GMS in NMDAR, are elevated in the postmortem brain tissue (51) and in the cerebrospinal fluid (CSF) of living schizophrenic patients (52), suggesting that GMS occupancy might be shifted toward antagonism in this disorder. Accordingly, modulation of NMDAR through the GMS has been proposed as a possible therapeutic target for the treatment of negative and cognitive symptoms in schizophrenia (53, 54).

Indeed, several agonists have been designed to either directly or indirectly target GMS due to its great potential for the treatment of negative and positive symptoms in schizophrenia. For example, 3-(4,6-dichloro-2-carboxyindol-3-yl) propionic acid, an indole-2-carboxylic acid derivative, has been found to have > 2,100-fold greater affinity for the GMS than glycine (55), and 3-hydroxy-imidazolidin-4-one derivatives are partial agonists of the GMS (56). Additional computational methods that can be used to identify potential agonists have been used (57). In addition to agonists of the GMS, GMS-specific antagonists, such as 7-chlorkynurenic or L-701,324, have been developed for research purposes (58, 59). Although numerous potential agonists and antagonists have been developed or identified, only a few of the candidates are suitable for advancement from preclinical studies to clinical trials. To date, most clinical studies have focused mainly on targeting the GMS using single amino acids as agonists of GMS, including glycine, D-cycloserine, and D-serine, as indicated in the bottom left panel of Figure 1.

### Direct Modulation of NMDAR Functions by Glycine

Glycine is the simplest amino acid and acts as a neurotransmitter in the CNS. In addition to glycinergic terminals, glycine may be simultaneously released into the synaptic cleft with GABA (60). Extracellular glycine is immediately recycled through glycine transporters, including glycine transporter 1 (GlyT1) in glial cells or glutamatergic neurons, and glycine transporter 2 (GlyT2) in presynaptic neurons (61). Intracellular glycine is then metabolized into L-serine by serine hydroxymethyltransferase in glial cells or catabolized into carbon dioxide and ammonium by the glycine cleavage system in neurons (62). Glycine causes inhibitory and excitatory neural transmission via strychnine-sensitive glycine receptors and NMDA receptors, respectively. Glycine receptors are mainly located in the brainstem and spinal cord. In contrast, NMDARs are present in high density within the cerebral cortices and hippocampus and are thought to be involved in the pathophysiology of schizophrenia (24, 63).

Numerous investigations support decreased glutamatergic signaling and NMDAR hypofunction as pathogenic mechanisms of schizophrenia. Interestingly, it has been reported that glycine is upregulated in patients with schizophrenia. Findings on schizophrenic patients obtained postmortem have revealed increased binding activity of radiolabeled [3H]glycine in the brain, especially in the parietal cortex and occipital cortex (64). Rats treated with a glycine-rich diet for a long period also exhibit schizophrenia-like abnormalities, including altered sensory gating function, enlarged cerebral ventricles, and diminished hippocampal dimensions (65). Similarly, high serum glycine levels have been reported in patients with chronic schizophrenia, and these levels have been associated with impaired sensorimotor gating function in pre-pulse inhibition (66). These findings imply that glycine levels might compensate for alterations in glutamate-NMDAR transmission in patients with chronic schizophrenia. For example, a postmortem study indicated a striking decrease in tyrosine phosphorylation of the GluN2 subunit in the dorsolateral prefrontal cortex of schizophrenic patients, but the postsynaptic density of NMDAR complexes in these patients was, in fact, increased (67). Inconsistently, lower plasma glycine levels have reported in schizophrenic patients compared to healthy controls and have been correlated with negative symptoms of schizophrenia (68). To further elucidate the glycine levels in the brains of schizophrenia patients, it is necessary to measure glycine levels in serum and CSF in a large sample size.

Despite the controversial findings regarding glycine levels in patients with schizophrenia, glycine-induced augmentation of NMDAR-mediated neurotransmission has been considered a potentially safe, and feasible approach for ameliorating negative symptoms of schizophrenia. Glycine appears to be safe, even at dosages of as high as 5 g/kg per day in rats (69) and 0.8 g/kg body weight per day in schizophrenic patients (70). In addition to its high biocompatibility and low toxicity, the effect of glycine on the amelioration of schizophrenia-related symptoms has been demonstrated in animal models of schizophrenia. Subchronic administration of glycine at doses relevant to its clinical effects (71) significantly prevents PCP-induced abnormalities in auditory mismatch negativity (MMN, a neurophysiological characteristic of schizophrenia) (72). Glycine also significantly reduced novelty- and methamphetamine-induced locomotor activity in neonatal ventral hippocampal damaged rats compared with sham rats (73). In addition, microinjection of 1 μmol of glycine into the mouse prefrontal cortex alleviated PCP-induced behavioral deficits in latent learning (74), suggesting the involvement of glycine in the regulation of frontocortical NMDARs and cognitive functions. Glycinamide, a prodrug of glycine, can be converted to glycine in CNS by hydrolysis and it prevented MK-801 (dizocilpine, a non-competitive antagonist of NMDAR)-induced deficits in a novel object recognition task in rabbits (75, 76). Despite contrasting neurochemical profiles, a recent study further proved that partial glycine site agonists and glycine reuptake inhibitors display comparable precognitive effects in rats and therefore have potential relevance as treatments of cognitive impairments in schizophrenia (77).

The effects of glycine on the treatment of schizophrenic symptoms in clinical studies are summarized in Table 1. Briefly, in the late 1980s, a series of open-label clinical studies failed to demonstrate the therapeutic potential of glycine in the amelioration of negative symptoms of schizophrenia (78–80). Milacemide, an acylated prodrug of glycine, did not alleviate schizophrenic symptoms, and psychotic symptoms were worsened (91, 92). Later, glycine was demonstrated to improve negative symptoms at 0.4 g/kg/day (81). Consistently, recent clinical studies have also indicated that a high dose of glycine is associated with improvement in clinical rating scales of schizophrenia, especially scales of negative symptoms (70, 71, 82, 83, 86, 89, 90). However, inconsistent results have been reported and indicate that glycine administered with clozapine had no effect on patients with schizophrenia (84, 85, 87). In a 16-week randomized double-blind, double-dummy, and parallel-group clinical trial conducted at four sites in the United States and one site in Israel, no significant differences were found between the total average scores on the Scale for the Assessment of Negative Symptoms (SANS) of patients treated with glycine or placebo, and no change in the average cognitive scores was apparent (88). The lack of consistency across trials could be due to small sample sizes, different doses of glycine, different trial durations, and different clinical ratings. Notably, glycine is an inhibitory neurotransmitter in glycinergic neurons, and it has been reported to have poor CNS penetration (i.e., rate of permeation across the blood-brain barrier) (93). Therefore, higher doses of glycine might be required for treatment purpose in patients. Unfortunately, systemic administration of high-does glycine is problematic and is not well-tolerated. The administration of high-dose glycine can result in some unwanted adverse effects, such as nausea (71, 83, 87) and sensorimotor gating deficits (94). Thus, these studies suggest that glycine is not a generally effective therapeutic option for treating negative symptoms or cognitive impairments. It seems wise to explore other drug candidates targeting GMS in the glutamatergic system.

**Table 1:** Summary of effects of glycine on the treatment of schizophrenic symptoms in clinical studies.

**Compound**	**Type**	**Study site**	**Patient**	**Usage**	**Subject number (placebo vs. experiment)**	**Dosage**	**Trial duration (weeks)**	**Clinical outcomes**	**Clinical ratings**	**References**
Glycine	OL	US	SZ	Add on	11 (no placebo)	5–25 (g/day)	32–36	–	Neuroleptics intake	(78)
	OL	US	SZ	Add on	6 (no placebo)	10.8 (g/day)	0.6–8	–	BPRS, SANS, CGI, SAS, AIMS	(79)
	OL	US	SZ	Add on	6 (no placebo)	15 (g/day)	6	–	BPRS	(80)
	DB + additional OL	US	SZ	Add on	7 vs. 7	2–30 (g/day)	8 DB + 8 OL	+ (Negative symptoms)	PANSS, ESRS, AIMS	(81)
	OL	US	SZ	Add on	5 (no placebo)	0.14–0.8 (g/kg/day)	8	+ (Negative symptoms)	PANSS, SANS, ESRS, AIMS	(82)
	DB (Crossover)	Israel	TRS SZ	Add on	11 vs. 11	0.8 (g/kg/day)	6	+ (Negative, depressive, cognitive symptoms)	PANSS, SAS, AIMS	(70)
	DB (Crossover)	Israel	TRS SZ	Add on	22 vs. 22	0.8 (g/kg/day)	6	+ (Negative, depressive, cognitive symptoms)	BPRS, PANSS, SAS, AIMS	(83)
	DB (Parallel)	US	TRS SZ	Add on (Clozapine)	10 vs. 9	30 (g/day)	12	–	BPRS, SANS, SAS, SAFTEE	(84)
	DB (Parallel)	US	SZ	Add on (Clozapine)	13 vs. 14	60 (g/day)	2 SB + 8 DB	–	BPRS, PNASS, SANS, HDRS, SAS, GAS	(85)
	DB (Crossover)	US	SZ	Add on	6 vs. 6	0.2–0.8 (g/kg/day)	6	+ (Negative symptoms)	PANSS, BARS, SAS, AIMS	(86)
	DB (Crossover)	Israel	SZ	Add on (Olanzapine & risperidone)	17 vs. 17 (Olanzapine: 12; Risperidone: 5)	0.06–0.8 (g/kg/day)	6	+ (Negative, cognitive, positive symptoms, excitement, depression)	BPRS, PANSS, SAS, AIMS	(71)
	DB (Crossover)	Canada	TRS SZ	Add on (Clozapine)	12 vs. 12	60 (g/day)	28	–	BPRS, PANSS, GAF, ESRS	(87)
	DB (Parallel) (NCT00222235)	US & Israel	SZ or SZA	Add on (Without clozapine)	45 (55) vs. 42 (54)	15–60 (g/day)	16	–	BPRS, SANS, CGI, SAS, AIMS	(88)
	DB (Parallel)	Australia	SZ or SZA	Add on	21 vs. 22 (SZ:17; SZA:5)	0.2–0.6 (g/kg/day)	6	+ (Acute: duration MMN; chronic:PANSS scores)	PANSS, CDRS, WSAS, ERP (MMN)	(89)
	DB (Crossover)	US	SZ (9p24.1 CNV)	Add on	2 vs. 2	6–48 (g/day)	6	+ (Clinical symptoms)	BPRS, PANSS, CGI, Motor abnormalities	(90)
	OL				2 (no placebo)	5.4–86.5 (g/day)	47	+ (Clinical symptoms)		

*+, Positive clinical results; −, Negative clinical results; AIMS, Abnormal Involuntary Movements Scale; BPRS, Brief Psychiatric Rating Scale; CDRS, Calgary Depression Rating Scale; CGI, Clinical Global Impression; DB, double-blind; ERP, Event Related Potential; ESRS, Extrapyramidal Symptom Rating Scale; GAF, Global Assessment of Functioning Scale; GAS, Global Assessment Scale; HDRS, Hamilton Depression Rating Scale; MMN, Mismatch negativity; OL, open-label; PANSS, Positive and Negative Syndrome Scale; SAFTEE, Systematic Assessment for Treatment Emergent Event; SANS, Scale for the Assessment of Negative Symptoms; SAS, Simpson Angus Scale for Assessment of Extrapyramidal Side Effects; SZ, schizophrenia; SZA, schizoaffective disorder; TRS: treatment-resistant; WSAS: Work and Social Adjustment Scale*.

### Direct Modulation of NMDAR Functions by D-Cycloserine

D-cycloserine is a well-known antibiotic metabolite produced by *Streptomyces orchidaceus* and *Streptomyces garyphalus* that has therapeutic effects on tuberculosis. D-cycloserine has also been found to act as a partial agonist targeting the GMS of NMDAR (95), and its binding affinity is 100-fold less than that of glycine (96). Similar to glycine, D-cycloserine has been reported to improve cognitive functions through modulation of NMDAR function in animal studies. For example, both systemic administration and intra-amygdala infusions of D-cycloserine facilitated conditioned fear extinction and improved memory consolidation in rats (97, 98). Single administration of D-cycloserine also significantly improved visual recognition memory in rhesus monkeys (99). However, inconsistently, some studies reported that D-cycloserine had no effect on neural activity in a mouse model of schizophrenia (100), MK-801-induced sensorimotor gating dysfunction in mice (101), or acquisition of memory performance in MK-801-treated rats in the radial arm maze and the water maze (102).

Similarly, inconsistent findings have also been reported in clinical studies. Effects of D-cycloserine on the treatment of schizophrenic symptoms in clinical studies are summarized in Table 2. Briefly, some studies indicated that D-cycloserine at a dosage of 50 or 100 mg/day had therapeutic effects in the treatment of negative symptoms and/or cognitive deficits (90, 103, 106, 108, 111, 112, 115–119). In contrast, others reported that D-cycloserine had no effect on patients with schizophrenia (88, 104, 107, 113, 114). There are several possible explanations for the contradictory findings in clinical studies. First, D-cycloserine has a very narrow therapeutic window. The administration of D-cycloserine >100 mg/day has been reported to result in the deterioration of clinical outcomes in patients with schizophrenia (96, 103, 110). It has been shown that D-cycloserine has neurotoxic side effects, including hyperexcitability, depression, anxiety, memory deficits, and even seizures (121). Second, D-cycloserine administered with clozapine can result in drug-drug interactions, which might lead to the exacerbation of symptoms in patients (105, 109). Third, the treatment effect of D-cycloserine might be influenced by heterogeneity caused by differences in onset age and white matter integrity (120). In addition, a study revealed that patients receiving D-cycloserine demonstrated a significant increase in temporal lobe activation, suggesting that the addition of D-cycloserine to conventional neuroleptics may improve negative symptoms through enhanced temporal lobe function (115). Finally, a meta-analysis indicated that full agonists (such as glycine and D-serine) appear to be more effective than partial agonists (such as D-cycloserine) (122, 123). Thus, the therapeutic potential of D-cycloserine appears to be limited and not particularly effective.

**Table 2:** Major findings in clinical trials examining effects of D-cycloserine on the treatment of schizophrenic symptoms.

**Compound**	**Type**	**Study site**	**Patient**	**Usage**	**Subject number (placebo vs. experiment)**	**Dosage**	**Trial duration (weeks)**	**Clinical outcomes**	**Clinical ratings**	**References**
D-cycloserine	OL	Italy	SZ	Add on	7 (No placebo)	250 (mg/day)	6	– (Worsen symptoms)	BPRS, SANS, CGI	(96)
	SB & RB (Dose finding)	US	SZ	Add on	9	5, 15, 50, 250 (mg/day)	10 (2 wks/dose)	+ (50 mg/day: negative, cognitive symptoms)	BPRS, SANS, GAS, SIRP, AIMS	(103)
	DB (Parallel)	US	SZ	Add on (Molindone)	4 vs. 3 vs. 6 (Placebo vs. 10 vs. 30)	10, 30 (mg/day)	4	–	BPRS, SANS, CGI	(104)
	SB & RB (Dose finding)	US	SZ	Add on (Clozapine)	10	5, 15, 50, 250 (mg/day)	10 (2 wks/dose)	– (Worsen symptoms)	BPRS, SANS, SIRP	(105)
	SB (Dose finding)	Netherlands	SZ (Drug-free)	Alone	13	15, 25, 50, 100, 250 (mg/day)	24 days (4 days/dose)	+ (100 mg/day: negative symptoms)	PANSS, CGI, ESRS	(106)
	DB (Crossover)	Israel	TRS SZ	Add on	8 vs. 9	50 (mg/day)	6	-	PANSS, HDRS, SAS, AIMS	(107)
	DB (Parallel)	US	SZ	Add on	23 (24) vs. 23 (23)	50 (mg/day)	8	+ (Nnegative symptoms)	PANSS, SANS, HDRS, GAS, SIRP, AIMS, Stroop Test, Miller-Selfridge Test, Verbal fluency, Digit span, Finger tapping	(108)
	DB (Crossover)	US	SZ	Add on (Clozapine)	11 vs. 11	50 (mg/day)	6	– (Worsen negative symptoms)	PANSS, SANS, HDRS, GAS, SAS, AIMS, BARS	(109)
	DB (Parallel)	Netherlands	SZ	Add on (Without antidepressants)	13:13	100 (mg/day)	8	– (Worsen symptoms)	PANSS, CGI, ESRS	(110)
	SB & RB (Dose finding)	US	SZ	Add on (Risperidone)	10	5, 15, 50, 250 (mg/day)	10 (2 wks/dose)	+ (50 mg/day: negative symptoms)	BPRS, SANS, HDRS, GAS, SAS, AIMS, Word list generation, Digit span, Finger tapping, Stroop test	(111)
	DB (Crossover)	Israel	TRS SZ	Add on	16 vs. 16	50 (mg/day)	6	+ (Negative symptoms)	PANSS,HDRS, SAS, AIMS	(112)
	DB (Parallel)	US	SZ	Add on	12 vs. 10	50 (mg/day)	4	–	BPRS, SANS, ATRS, SAS, CPT, Sternberg paradigm	(113)
	DB (Parallel)	US	SZ	Add on	12 (28) vs. 14 (27)	50 (mg/day)	24	–	PANSS, SANS, HDRS, QOL, GAS, CVLT, WAIS III, ANART, Stroop Test, Finger tapping, WCST, SAS, AIMS	(114)
	DB (Parallel)	US	SZ	Add on	6 vs. 6	50 (mg/day)	8	+ (Improved negative symptoms associated with temporal lobe activation)	PANSS, SANS, SAS, AIMS, fMRI	(115)
	DB (Parallel)	US & Israel	SZ or SZA	Add on (Without cloazpine)	45 (55) vs. 46 (56)	25–50 (mg/day)	16	–	BPRS, SANS, CGI, SAS, AIMS	(88)
	DB (Parallel)	US	SZ	Add on (Without cloazpine)	16 (19) vs. 16 (19)	50 (mg/day)	8	+ (Negative symptmos, logical memory)	PANSS, SANS, CGI, SAFTEE, WMS-III, HVLT, WCST, TMT, Phonemic fluency, Category fluency, Letter-number sequencing, Grooved pegboard	(116)
	DB (Crossover) (NCT00742079)	US	SZ or SZA	Add on (Combined with CBT)	9 (10) vs. 11 (11) (PCB-first vs. DCS-first)	50 (mg/day)	1	+ (DCB-first: delusional severity, distress, belief conviction)	PSYRATS, SAPS, ABA, Bead Task	(117)
	DB (Parallel) (NCT00963924)	US	SZ or SZA	Add on	15 (18) vs. 17 (18)	50 (mg/day)	8	+ (Cognitive, negative symptoms)	PANSS, SANS, MATRICS, CDSS, QOL, GAS, SAFTEE, Auditory discrimination task	(118)
	DB (Parallel)	US	SZ	Add on	21 vs. 24	100 (mg/once)	1 day	+ (Neural response, working memory)	BPRS, WASI, EEG, N-back task, IIT, WPT	(119)
	DB (Crossover) (UMIN000000468)	Japan	SZ	Add on	19 (22) vs. 17 (19) (PCB-first vs. DCS-first)	50 (mg/day)	6	–	PANSS, SANS, BACS, JCDSS, GAF, EQS, DIEPSS, AIMS, MR-DTI	(120)
	DB (Crossover)	US	SZ (9p24.1 CNV)	Add on	2 vs. 2	50 (mg/day)	6	+ (Clinical symptoms)	BPRS, PANSS, CGI, Motor abnormalities	(90)

*+, Positive clinical results; −, Negative clinical results; ABA, Alternative Beliefs Assessment; AIMS, Abnormal Involuntary Movements Scale; ANART, Adult North American Reading Test; ATRS, Abrams and Taylor Rating Scale; BACS, Brief Assessment of Cognition in Schizophrenia; BARS, Barnes Akathisia Rating Scale; BPRS, Brief Psychiatric Rating Scale; CDSS, Calgary Depression Scale of Schizophrenia; CGI, Clinical Global Impression; CPT, Continuous Performance Test; CVLT, California Verbal Learning Test; DB, double-blind; DCS, D-cycloserine; DIEPSS, Drug Induced Extrapyramidal Symptoms Scale; DTI, Diffusion Tensor Imaging; EEG, electroencephalogram; EQS, Emotional Intelligence Scale; ESRS, Extrapyramidal Symptom Rating Scale; fMRI, functional Magnetic Resonance Imaging; GAF, Global Assessment of Functioning Scale; GAS, Global Assessment Scale; HDRS, Hamilton Depression Rating Scale; HVLT, Hopkins Verbal Learning Test; IIT, Information Integration Task; JCDSS, Japanese version of Calgary Depression Scale of Schizophrenia; MATRICS, Measurement and Treatment Research to Improve Cognition in Schizophrenia; OL, open-label; PANSS, Positive and Negative Syndrome Scale; PSYRATS, Psychotic Symptom Rating Scales; QOL, Quality of Life; RB, rater-blind; SAFTEE, Systematic Assessment for Treatment Emergent Event; SANS, Scale for the Assessment of Negative Symptoms; SAPS, Assessment of Positive Symptoms; SAS, Simpson Angus Scale for Assessment of Extrapyramidal Side Effects; SB, single-blind; SIRP, Sternherg's Item Recognition Paradigm; SZ, schizophrenia; SZA, schizoaffective disorder; TMT, Trail Making Test; TRS, treatment-resistant; WAIS-III, Wechsler Adult Intelligence Scale-III; WASI, Weschler Abbreviated Scale of Intelligence; WCST, Wisconsin Card Sorting Test; WPT, Weather Prediction Task*.

### Direct Modulation of NMDAR Functions by D-Serine

D-serine is enriched in the forebrain and is an endogenous ligand of the GMS on NMDAR (124). Emerging evidence suggests the potential role of D-serine in the regulation of NMDAR functions for the treatment of schizophrenia. For the GluN1/N2 subunits of NMDAR, the binding affinity of D-serine is three-fold more potent than that of glycine (125). D-serine is mainly expressed by glutamatergic neurons, even though there has been considerable controversy regarding the concentration and function of D-serine in glial cells and neurons (126). D-serine is predominantly produced in neurons by the stereoconversion of L-serine (provided by astrocytes) via the PLP-dependent enzyme serine racemase (SR) and is then shuttled to astrocytes, where it is stored and released. Studies using more-selective antibodies have demonstrated that SR and D-serine are prominently expressed in forebrain glutamatergic neurons (127–130). In addition, the distribution of D-serine residues in the brain is similar to that of NMDARs (131). Intriguingly, it has been reported that the deletion of neuronal SR resulted in impaired NMDAR functions and synaptic plasticity, whereas deletion of astrocytic SR had no effect (132). Notably, D-serine is the primary coagonist of synaptic NMDARs, whereas glycine is the primary coagonist of extrasynaptic NMDARs (22). In general, D-serine is an allosteric modulator of brain NMDARs and is predominantly released from glutamatergic neurons.

Emerging evidence suggests that D-serine is involved in the pathophysiology of schizophrenia and is a potential therapeutic agent and/or biomarker for schizophrenia. Indeed, decreased levels of D-serine in serum and CSF have been found in patients with schizophrenia compared to those in healthy controls (133). A CSF and postmortem brain study also revealed a 25% decreases in D-serine levels and the D/L-serine ratio in the CSF of schizophrenia patients, suggesting that reduced brain SR and elevated D-amino acid oxidase (DAO) protein levels may contribute to the lower D-serine levels observed in the CSF of schizophrenic patients (28). A recent study further indicated that poor executive function performance is associated with a lower D-serine/total serine ratio in schizophrenic patients (134). Moreover, accumulating evidence has indicated that alteration of D-serine is associated with neuroplasticity and cognitive deficits in schizophrenia. For example, supplementation with D-serine prevented the onset of cognitive deficits in adult offspring after maternal immune activation in pregnant mice (135), suggesting that early intervention with D-serine may prevent the occurrence of psychosis in high-risk subjects. Decreasing synaptic D-serine by enhancing Na^+^-independent alanine–serine–cysteine transporter-1 abolished long-term potentiation (LTP) and reduced synaptic NMDAR responses by 60–70% (136). Taking advantage of SR-null mice, a series of studies confirmed that D-serine is required for NMDAR responses, NMDAR-dependent LTP, dendritic spine formation, cognitive functions, and social memory (137–141). However, D-serine is metabolized rapidly by DAO, reducing its bioavailability and requiring the administration of high doses, which may lead to peripheral neuropathies, creating a potential problem for the use of D-serine in treating schizophrenia-related symptoms (142, 143). D-serine levels in blood and urine are sensitive to the presence of kidney dysfunction of different origins. There are also concerns that high concentrations of D-serine augment kidney dysfunction and cause potential nephrotoxicity, which has been reported in rats that have developed acute tubular necrosis associated with higher doses of D-serine (144, 145). Nevertheless, serum D/L-serine levels might provide a measurable biological marker for schizophrenia, and D-serine may be effective for the treatment of negative symptoms and cognitive dysfunction in schizoprhenia. The study of D-serine requires accurate methodologies and specific controls, and a specific guideline for accurate measurement and detection methods has been described previously (146).

Along the same lines, D-serine has been employed alone or as an add-on treatment to standard antipsychotics for improving positive, negative, and cognitive symptoms of schizophrenia in numerous clinical studies (147–159). Effects of D-serine on the treatment of schizophrenic symptoms in clinical studies are summarized in Table 3. Briefly, some clinical studies have demonstrated positive outcomes for D-serine (147, 149–151), and repeated D-serine administrations have been shown to improve MMN and cortical plasticity in patients with schizophrenia (156, 157). However, other studies have revealed negative results (148, 152–155). A meta-analysis indicated that the effect size of D-serine on the treatment of negative symptoms (SMD = −0.319) and positive symptoms (SMD = −0.211) appeared to be small (160). In particular, in the first randomized double-blind placebo-controlled study with 60 mg/kg D-serine in schizophrenia, D-serine led to significant improvement in MMN frequency generation and clinical symptoms (157), which is consistent with another meta-analyses showing significant effects of D-serine on schizophrenia. This study also implied that a minimum daily dose of 3.6 g D-serine is needed to improve negative symptoms. However, high concentrations of D-serine can lead to peripheral neuropathies, such as oxidative damage (161), neurotoxicity (162), and renal toxicity (150, 163). In summary, these studies indicate that the therapeutic benefit of D-serine may be limited due to its adverse effects.

**Table 3:** Summary of clinical outcomes and benefits related to D-serine in patients with schizophrenia.

**Compound**	**Type**	**Study site**	**Patient**	**Usage**	**Subject number (placebo vs. experiment)**	**Dosage**	**Trial duration (weeks)**	**Clinical outcomes**	**Clinical ratings**	**References**
D-serine	DB (Parallel)	Taiwan	SZ	Add on	15 vs. 14	30 (mg/kg/day)	6	+ (Positive, negative, cognitive symptoms)	PANSS, SANS, CGI, HDRS, SAS, AIMS, BARS, UKU	(149)
	DB (Parallel)	Taiwan	SZ	Add on (Clozapine)	10 vs. 10	30 (mg/kg/day)	6	–	PANSS, SANS, CGI, HDRS, SAS, AIMS, BARS, UKU	(152)
	DB (Crossover)	Israel	TRS SZ	Add on (Olanzapine & risperidone)	38 vs. 37 (Risperidone: 21; Olanzapine: 18)	20–30 (mg/kg/day)	6	+ (Negative, positive, cognitive, depression symptoms)	BPRS, PANSS, SANS, SAS, AIMS	(147)
	DB (Parallel)	Taiwan	SZ (Acute exacerbation)	Add on (Risperidone)	20 (23) vs. 19 (21)	2 (g/day)	6	–	PANSS, SANS, SAS, AIMS, BARS, UKU	(149)
	DB (Parallel) (NCT00491569)	Taiwan	SZ	Add on	16 (20) vs. 16 (20)	2 (g/day)	6	–	PANSS, SANS, GAF,QOL, SAS, AIMS, BARS, UKU	(153)
	OL (NCT00322023)	US	SZ or SZA	Add on (Without cloazpine)	12 vs. 19 vs. 16 (30 vs. 60 vs. 120; no placebo)	30, 60, 120 (mg/kg/day)	4	+ (PANSS, MATRICS, neuropsychological measures)	PANSS, SANS, CGI, CDSS MATRICS, SAS, AIMS, BARS	(150)
	DB (Parallel) (NCT00138775)	Israel	SZ or SZA	Add on	69 (98) vs. 73 (97)	2 (g/day)	16	–	PANSS, SANS, CGI, SAS, AIMS, UKU	(154)
	DB (Parallel)	Israel	TRS SZ	Alone	5 (10) vs. 3 (8) (D-serine vs. Olanzapine)	1.5–3 (g/day)	10	Treatment effect: Olanzapine > D-serine	PANSS, SAS, AIMS, UKU	(158)
	DB (Parallel)	US & India	SZ or SZA	Add on	23 (26) vs. 25 (27) vs. 22 (27) vs. 21 (24) (control vs. D-serine vs. CRT vs. D-serine + CRT)	30 (mg/kg/day)	12	–	PANSS, CDS, QOL, CPT, WAIS-III, HVLT-R, TOL, WCST, SAS, AIMS, BARS, UKU	(155)
	OL	Israel	TRS SZ	Add on	17 (no placebo)	1.5–4 (g/day)	6	+ (Extreme delta brush electrographic pattern)	MRI, continuous EEG	(159)
	DB (Parallel) (NCT00826202)	US	SZ Prodrome	Add on	20 (24) vs. 15 (20)	60 (mg/kg/day)	16	+ (Negative symptoms)	SOPS, MATRICS, PSQI, SAS, AIMS, SAFTEE	(151)
	DB (Crossover) (NCT01474395)	US	SZ or SZA	Add on	13 (one placebo session + two D-serine sessions)	60 (mg/kg/day)	2–3	+ (Auditory plasticity, θ-frequency response, MMN generation)	Auditory emotion paradigm, ERP(MMN)	(156)
	OL (NCT02156908)				3 vs. 5					
	DB (Crossover) (NCT00817336)	US	SZ or SZA	Add on	16 vs. 16	60 (mg/kg/day)	6	+ (MMN frequency, generation, clinical symptoms)	PANSS, MCCB, ERP (MMN)	(157)
	OL (NCT00322023)		SZ or SZA		5 vs. 8 vs. 6 (30 vs. 60 vs. 120; no placebo)	30, 60, 120 (mg/kg/day)	4	+ (MMN frequency)		

*+, Positive clinical results; –, Negative clinical results; AIMS, Abnormal Involuntary Movements Scale; ANSS, Positive and Negative Syndrome Scale; BARS, Barnes Akathisia Rating Scale; BPRS, Brief Psychiatric Rating Scale; CDS, Calgary Depression Scale; CDSS, Calgary Depression Scale of Schizophrenia; CGI, Clinical Global Impression; CPT, Continuous Performance Test; DB, double-blind; EEG, Electroencephalogram; ERP, Event Related Potential; GAF, Global Assessment of Functioning Scale; HDRS, Hamilton Depression Rating Scale; HVLT-R, Hopkins Verbal Learning Test-Revised; MATRICS, Measurement and Treatment Research to Improve Cognition in Schizophrenia; MCCB, MATRICS consensus cognitive battery; MMN, Mismatch negativity; MRI, Magnetic Resonance Imaging; OL, open-label; PSQI, Pittsburgh Sleep Quality Index; QOL, Quality of Life; SAFTEE, Systematic Assessment for Treatment Emergent Event; SAS, Simpson Angus Scale for Assessment of Extrapyramidal Side Effects; SANS, Scale for the Assessment of Negative Symptoms; SOPS, Scale of Prodromal Symptoms; SZ, schizophrenia; SZA, schizoaffective disorder; TOL, Tower of London Test; TRS, treatment-resistant; UKU, Udvalg for Kliniske Undersogelser Side Effects Rating Scale; WAIS-III, Wechsler Adult Intelligence Scale-III; WCST, Wisconsin Card Sorting Test*.

## Indirectly Targeting the GMS on NMDARs

As described previously, activation of NMDARs requires the binding of a coagonist, D-serine or glycine, at the GMS of NMDARs. To date, the GMS on NMDAR is one of the most promising therapeutic targets for contributing to the medical needs of patients with schizophrenia. However, the beneficial effect of directly targeting the GMS with D-serine is limited because of the requirements for a high dose, narrow therapeutic window and poor CNS penetration rate, concomitant side effects and potential drug-drug interactions. Alternatively, as illustrated in the right panel of Figure 1, indirectly targeting the GMS of NMDARs via enhancement of synaptic glycine/D-serine levels from in astrocytes provides a new approach to modulate NMDAR functions and to help meet the needs of patients in schizophrenia (164).

### Indirect Modulation of NMDAR Functions by Targeting Astrocytic GlyT1

A glycine reuptake inhibitor inhibits the reuptake of synaptic glycine by blocking astrocytic glycine transporters and increasing the availability of glycine at the synaptic cleft. Glycine transporter type 1 (GlyT1) is expressed at glutamatergic synapses throughout mammalian brain regions and primarily regulates the synaptic concentrations of glycine (165). GlyT1 is highly colocalized with NMDARs on glial cells and neurons in the cortex, hippocampus, septum and thalamus (166). GlyT1 effectively regulates synaptic glycine reuptake and governs GMS occupancy at NMDARs in excitatory synapses (19). Thus, selective inhibition of astrocytic GlyT1 is a promising new therapeutic target for indirectly enhancing synaptic glycine concentrations and facilitating NMDAR function.

Accumulating evidence from preclinical studies indicates that inhibition of GlyT1 enhances NMDAR functions in animals. Initial studies have revealed that glycyldodecylamide, a non-selective glycine transport antagonist, reverses PCP-induced behavioral deficits (167, 168). Subsequently, a series of studies consistently demonstrated that administration of N[3-(40-fluorophenyl)-3-(40-phenylphenoxy)propyl]-sarcosine (NFPS, also known as Alx5470), a GlyT1 inhibitor, enhanced LTP and behavioral performances in associative learning, spatial and object memory, and social memory (140, 141, 169–171). In agreement with the results obtained with NFPS, a series of studies also indicated that sarcosine, another GlyT1 inhibitor, has promising therapeutic potential in ameliorating behavioral impairments and cognitive deficits in both pharmacological and genetic mouse models of schizophrenia (139, 172, 173). Furthermore, sarcosine has been proven to effectively regulate the surface trafficking of NMDARs, NMDAR-evoked electrophysiological activity, brain glycine levels and MK-801-induced abnormalities in the brain, which might contribute to the therapeutic effect for the treatment of schizophrenia (139). Intriguingly, it has been proven that sarcosine also binds to the GMS of NMDARs and enhances NMDAR functions through more than one mechanism (139, 174). In addition, other GlyT1 inhibitors, such as SSR504734 and ORG 24598, have also displayed similar beneficial effects in sensorimotor gating, learning and memory functions, and schizophrenia-like behaviors (175–178). Furthermore, selective genetic disruption of GlyT1 resulted in enhancement of NMDAR functions, spatial retention memory, selective attention, and procognitive and antipsychotic phenotypic profiles, suggesting that inhibition of GlyT1 might have both cognitive-enhancing and antipsychotic effects (179–181). These studies indicate that GlyT1 is an attractive and promising drug target for the treatment of schizophrenia-related behaviors and cognitive deficits, even though the high binding affinity of the GlyT1 inhibitor can cause unpredictable toxicity leading to a coma-like state, compulsive walking or respiratory distress (15, 182).

With the aim of treating unmet medical needs in schizophrenia, a number of pharmaceutical industries have developed selective GlyT1 inhibitors as novel therapeutic drugs for schizophrenia. Numerous clinical studies have been carried out to evaluate the effects of special GlyT1 inhibitors on the treatment of schizophrenic symptoms. Based on the chemical structures of GlyT1 inhibitors, these clinical studies can be divided into two major structural classes: sarcosine-based and non-sarcosine-based inhibitors, and the summaries of these studies are shown in Tables 4, 5, respectively.

**Table 4:** Summary of clinical trials evaluating effects of sarcosine-based GlyT1 inhibitors on the treatment of schizophrenic symptoms.

**Compound**	**Type**	**Study site**	**Patient**	**Usage**	**Subject number (placebo vs. experiment)**	**Dosage**	**Trial duration (weeks)**	**Clinical outcomes**	**Clinical ratings**	**References**
Org 25935	DB (Parallel) (NCT00725075)	Worldwide (GINAT trial)	SZ (Negative symptom)	Add on	62 (70) vs. 62 (71) vs. 67 (73) (Placebo vs. low-dose vs. high-dose)	4–8 & 12-16 (mg, BID)	12	–	PANSS, SANS, GAF, CDSS, NES, Cognitive battery, ESRS	(183)
AMG 747	DB (Parallel) (NCT01568216 & NCT01568229)	Worldwide	SZ	Add on	76 (90) vs. 54 (60) vs. 51 (60) vs. 51 (60) (placebo vs. 5 vs. 15 vs. 40)	5, 15, 40 (mg/day)	12	Terminated (Adverse event)	PANSS, NSA-16, CGI, MCCB, PSP, Q-LES-Q-18, SDS	(184)
Sarcosine	DB (Parallel)	Taiwan	SZ	Add on	21 vs. 17	2 (g/day)	6	+ (Positive, negative, cognitive, gnenral symptoms)	BPRS, PANSS, SANS, HDRS, SAS, AIMS, BARS, UKU	(185)
	DB (Parallel)	Taiwan	SZ (Acute exacerbation)	Add on (Risperidone)	20 (23) vs. 18 (21)	2 (g/day)	6	+ (Positive, negative symptoms)	PANSS, SANS, SAS, AIMS, BARS, UKU	(148)
	DB (Parallel)	Taiwan	TRS SZ	Add on (Clozapine)	10:10	2 (g/day)	6	–	PANSS, SAS, AIMS, BARS, UKU	(186)
	DB (Parallel) (NCT00328276)	Taiwan	SZ (Drug-free) (Acute exacerbation)	Alone	6 (9) vs. 10 (11) (1 vs. 2; no placebo)	1, 2 (g/day)	6	–	PANSS, SANS, QOL, SAS, AIMS, BARS, UKU	(187)
	DB (Parallel) (NCT00491569)	Taiwan	SZ	Add on	16 (20) vs. 19 (20)	2 (g/day)	6	+ (Positive, negative symptoms)	PANSS, SANS, GAF, QOL, SAS, AIMS, BARS, UKU	(153)
	OL (Case report)	Poland	SZ	Add on (Quetiapine and citalopram)	1	1, 2 (g/day)	4 (2 g/day: 2 + 1 g/day: 2)	+ (2 g: negative symptom but cause hypomania)	PANSS, HDRS	(188)
	OL (Case report)	Poland	SZ (Negative/cognitive symptoms)	Add on (Olanzapine and venlafaxine)	1	2 (g/day)	12 (24)	Terminated (Cause hypomania)	PANSS, HDRS	(189)
	DB (Parallel) (NCT01503359)	Poland (PULSAR)	SZ (Negative symptom)	Add on	25 vs. 25	2 (g/day)	24	– (Negative, general symptoms) (Decreased in hippocampal Glx/Cr, Glx/Cho)	PANSS, 1H-MRS	(190)
			Paranoid SZ	Add on	29 vs. 30	2 (g/day)	24	No changes of cardiometabolic & body composition parameters	PNASS, BIA, Cardiometabolic characteristics	(191)
			SZ (Negative symptom)	Add on	25 vs. 25	2 (g/day)	24	+ (Negative symptom) (Increased in DLPFC NAA/Cho, mI/Cho, mI/Cr)	PANSS, 1H-MRS	(192)
			SZ (Negative symptom)	Add on	25 vs. 25	2 (g/day)	24	+ (Negative symptom) (Decreased in WM Glx/Cr. Glx/Cho)	PANSS, 1H-MRS	(193)
			Paranoid SZ	Add on	30 vs. 28	2 (g/day)	24	+ (Negative, total symptoms) (MMP-9 no changed)	PANSS, CDSS, BIA serum MMP-9 measure	(194)
			Paranoid SZ	Add on	30 vs. 27	2 (g/day)	24	+ (Negative, total symptoms) (BDNF no changed)	PANSS, CDSS, BIA Serum BDNF measure	(195)
			SZ (Negative symptom)	Add on	29 vs. 27	2 (g/day)	24	+ (Negative, total symptoms) (IL-6 no changed)	PANSS, CDSS, BIA Serum IL-6 measure	(196)
			Paranoid SZ	Add on	29 vs. 27	2 (g/day)	24	+ (Negative, total symptoms) (TNFα no changed)	PANSS, CDSS, BIA Serum TNFα measure	(197)
	OL	Israel	SZ or SZA	Add on	5 vs. 5	2 (g/day)	1	No result (Sample size too small)	PANSS, CGI, MCCB, CDSS, SAS, AIMS	(198)
					17 vs. 17	4 (g/day)		+ (Positive, general symptoms)		
	DB (Parallel)	Taiwan	SZ	Add on	16 (21) vs. 16 (21) (Placebo vs. sarcosine)	2 (g/day)	12	–	PANSS, CGI, GAF, MCCB, SAS, AIMS, BARS, UKU	(199)

*+, Positive clinical results; –, Negative clinical results; 1H-MRS, Proton magnetic resonance spectroscopy; AIMS, Abnormal Involuntary Movements Scale; BARS, Barnes Akathisia Rating Scale; BDNF, Brain-derived neurotrophic factor; BIA, Bioelectrical Impedance Analysis; BPRS, Brief Psychiatric Rating Scale; CDSS, Calgary Depression Scale of Schizophrenia; CGI, Clinical Global Impression; Cho, Choline; Cr, Creatine; DB, double-blind; ESRS, Extrapyramidal Symptom Rating Scale; GAF, Global Assessment of Function; Glx, Complex of glutamate, glutamine and GABA; HDRS, Hamilton Depression Rating Scale; IL-6, Interleukin-6; MCCB, MATRICS consensus cognitive battery; mI, Myo-inositol; MMP-9, Matrix metallopeptidase-9; NAA, N-acetylaspartate; NES, Neurological Evaluation Scale; NSA-16, Negative Symptom Assessment-16; OL, open-label; PANSS, Positive and Negative Syndrome Scale; PSP, Personal and Social Performance Scale; Q-LES-Q-18, Quality of Life Enjoyment and Satisfaction Questionnaire; QOL, Quality of Life; SANS, Scale for the Assessment of Negative Symptoms; SAS, Simpson Angus Scale for Assessment of Extrapyramidal Side Effects; SDS, Sheehan Disability Scale; SZ, schizophrenia; SZA, schizoaffective disorder; TNFα, Tumor necrosis factor α; UKU, Udvalg for Kliniske Undersogelser Side Effects Rating Scale; WM, White matter*.

**Table 5:** Major findings in clinical trials examining effects of non-sarcosine-non-sarcosine-based glycine transporter 1 (GlyT1) inhibitors in patients with schizophrenia.

**Compound**	**Type**	**Study site**	**Patient**	**Usage**	**Subject number (placebo vs. experiment)**	**Dosage**	**Trial duration (weeks)**	**Clinical outcomes**	**Clinical ratings**	**References**
SSR504734	Phase I		SZ		Undisclosed details			Terminated		(182)
SSR103800	Phase I		SZ		Undisclosed details			Terminated		(182)
GSK1018921	DB (Parallel) (NCT00929370)		SZ		Undisclosed details		4	Terminated	PANSS, CGI, VAS, SAS, AIMS, BARS	(200)
DCCCyB	Phase I		SZ		Undisclosed details			Terminated		(182)
PF-03463275	DB (Crossover) (NCT01911676)	US	SZ	Add on (Risperidone, aripiprazole)	9 (12) (Risperidone: 5 (6), aripiprazole: 4 (6))	10, 20, 40 (mg, BID)	1	+ (40 mg: enhanced neuroplasticity)	PET, EEG (LTP)	(201)
				Add on	10 (11)	60 (mg, BID)	1	–		(202)
	DB (Parallel) (NCT00977522)	US	SZ (Negative Symptom)	Add on	207 (Total)	30 (mg, BID)	12	Teminated	PANSS, SANS, CGI, GAS, MCCB, SQLS, C-SSRS, ESRS	(203)
Bitopertin	DB (Parallel) (NCT01192867)	Worldwide (FlashLyte)	SZ (Negative Symptom)	Add on	594 (total)	10, 20 (mg/day)	24	–	PANSS, CGI, PSP	(204)
	DB (Parallel)	Worldwide (CandleLyte)	SZ (Acute exacerbation)	Alone	58 (80) vs. 56 (80) vs. 60 (77) (Placebo vs. 10 vs. 30)	10, 30 (mg/day)	4	–	PANSS, CGI, C-SSRS, SCID-CT, ESRS, NOSIE, ESRS	(205)
	DB (Parallel) (NCT01192906)	Worldwide (DayLyte)	SZ (Negative Symptom)	Add on	605 (Total)	5, 10 (mg/day)	24	–	PANSS, PSP	(206)
	DB (Parallel) (NCT00616798)	Worldwide	SZ (Negative/ disorganized thought)	Add on	61 (81) vs. 60 (82) vs. 57 (81) vs. 53 (79) (Placebo vs. 10 vs. 30 vs. 60)	10, 30, 60 (mg/day)	8	+ (Negative symptoms)	PANSS, CGI, PSP, SQLS, HRQoL, SAS, AIMS, BARS	(207)
										(208)
								– (Quality of life)		(209)
	DB (Parallel) (JapicCTI-111627)	Japan	SZ (Negative Symptom)	Add on	9 (15) vs. 57 (73) vs. 48 (73) (No placebo)	5, 10, 20 (mg/day)	52	+ (Negative & sub-optimally controlled symptoms) (20 mg: adverse events)	PANSS, CGI, PSP, C-SSRS, ESRS	(210)
	DB (Parallel) (NCT01235520)	Worldwide (TwiLyte)	SZ	Add on	186 (196) vs. 188 (198) vs. 186 (194) (Placebo vs. 10 vs. 20)	10, 20 (mg/day)	12	–	PANSS, CGI, PSP, C-SSRS, ESRS	(211)
	DB (Parallel) (NCT01235585)	Worldwide (MoonLyte)			186 (193) vs. 187 (195) vs. 191 (200) (Placebo vs. 5 vs. 10)	5, 10 (mg/day)		–		
	DB (Parallel) (NCT01235559)	Worldwide (NightLyte)			189 (199) vs. 190 (198) vs. 190 (199) (Placebo vs. 10 vs. 20)	10, 20 (mg/day)		+ (10 mg: positvie symptoms)		
	DB (Parallel) (NCT01192880)	Worldwide (SunLyte)	SZ (Negative Sympt)	Add on	625 (630)	10, 20 (mg/day)	24	– (Small Effect size)	PANSS, NSA-16, CGI, PSP, C-SSRS, ESRS	(212)
	DB (Parallel) (NCT01192906)	Worldwide (DayLyte)			203 (209) vs. 205 (211) vs. 197 (201) (Placebol vs. 5 vs. 10)	5, 10 (mg/day)		–		
	DB (Parallel) (NCT01192867)	Worldwide (FlashLyte)			197 (210) vs. 200 (208) vs. 197 (208) (Placebo vs. 10 vs. 20)	10, 20 (mg/day)		–		
	DB (Parallel) (NCT01116830)	US	SZ or SZA	Add on	12 vs. 17	10 (mg/day)	6	–	PANSS, MCCB, ERP (MMN)	(213)
	OL (NCT01116830)	US	SZ or SZA	Add on	12 vs. 17	10 (mg/day)	6	–	PANSS, MCCB, ERP (MMN)	(157)
BI 425809	DB (Parallel) (NCT03859973)	Worldwide	SZ	Add on (without clozapine)	200 (Total)	10 (mg/day)	12	Recruiting	PANSS, CGI, MCCB, SCoRS, BET, VRFCAT, PRECIS	(214)
	DB (Parallel) (NCT02832037)	Worldwide	SZ	Add on	160 (170) vs. 77 (85) vs. 79 (84) vs. 81 (85) vs. 83 (85) (Placebo vs. 2 vs. 5 vs. 10 vs. 25)	2, 5, 10, 25 (mg/day)	12	+ (Cognitive symptoms)	PANSS, MCCB, PSP, SCoRS, C-SSRS	(215)

*+, Positive clinical results; –, Negative clinical results; AIMS, Abnormal Involuntary Movements Scale; BARS, Barnes Akathisia Rating Scale; BET, Balloon Effort Task; CGI, Clinical Global Impression; C-SSRS, Columbia-Suicide Severity Rating Scale; DB, double-blind; EEG, Electroencephalogram; ERP, Event Related Potential; ESRS, Extrapyramidal Symptom Rating Scale; GAS, Global Assessment Scale; HRQoL, Health-Related Quality of Life; LTP, Long-term potentiation; MCCB, MATRICS consensus cognitive battery; MMN, Mismatch negativity; NOSIE, Nurses' Observation Scale for Inpatient Evaluation; NSA-16, Negative Symptom Assessment-16; OL, open-label; PANSS, Positive and Negative Syndrome Scale; PRECIS, Patient Reported Experience of Cognitive Impairment in Schizophrenia; PSP, Personal and Social Performance Scale; SANS, Scale for the Assessment of Negative Symptoms; SAS, Simpson Angus Scale for Assessment of Extrapyramidal Side Effects; SCID-CT, Structured Clinical Interview for DSM-IV–Clinical Trials version; SCoRS, Schizophrenia Cognition Rating Scale; SQLS, Schizophrenia Quality of Life Scale; SZ, schizophrenia; SZA, schizoaffective disorder; VAS, Visual Assessment Scale; VRFCAT, Virtual Reality Functional Capacity Assessment Tool*.

#### Sarcosine-Based GlyT1 Inhibitors

In the early period of drug discovery, several high-affinity GlyT1 inhibitors derived from sarcosine derivatives [e.g., NFPS (141, 169, 177) and Org 24598 (178)] were produced but caused unexpected toxicity and side effects (15, 178). Only two sarcosine-based GlyT1 inhibitors, AMG 747 (184) and Org 25935 (also known as SCH 900435 or MK-8435) (183), were advanced into clinical trials. Both AMG 747 and Org 25935 trials ended due to unspecified safety events and failure to benefit schizophrenia, respectively (182). Researchers have focused on the low-affinity GlyT1 inhibitor sarcosine as an adjunctive medication to conventional antipsychotics. Off-label use of sarcosine in clinical studies has been demonstrated to improve positive symptoms, negative symptoms, and quality of life with minimal side effects in patients with schizophrenia (148, 153, 185, 198). Moreover, findings from previous clinical trials and moderator analyses further indicated that sarcosine is more efficacious than D-serine in general psychopathology for chronically ill stable schizophrenic patients as well as for schizophrenic patients with acutely exacerbated symptoms of schizophrenia (123, 148, 153). Along the same lines, a series of studies from the Polish Sarcosine Study in Schizophrenia (PULSAR) project illustrated that schizophrenic patients treated with sarcosine for 6 months displayed significant improvements in negative symptoms, general psychopathology and changes in glutamatergic transmission in the brain (190, 192, 193). However, no significant differences in cardiometabolic systems, body composition or neurochemical levels (e.g., BDNF, IL-6 and TNF-α) were found in PULSAR studies (191, 194–197). Double-blind clinical studies revealed no beneficial effect of adjunctive sarcosine in drug-free schizophrenia patients or patients treated with clozapine (186, 187, 199). In terms of the side effects and safety profile of sarcosine, the overall results have been satisfactory in most clinical studies; however, sarcosine administered with glutamatergic and serotoninergic agents may have had a synergistic effect that exacerbated schizophrenic symptoms and hypomania in two case reports (188, 189).

#### Non-sarcosine-based GlyT1 Inhibitors

In addition to sarcosine-based inhibitors, non-sarcosine-derived GlyT1 inhibitors are potential alternatives for indirectly modulating the GMS on NMDARs. Compared to sarcosine-based GlyT1 inhibitors, non-sarcosine-based compounds are associated with faster off-rates and less toxic side effects (182). The earliest non-sarcosine-based GlyT1 inhibitors, including SSR504734 (216), SSR103800 (217), GSK1018921 (218), and DCCCyB (219), were developed and have been entered into phase I clinical trials. However, the trials with all these compounds were halted or discontinued for undisclosed reasons (182, 200, 220). In addition, PF-3463275, another non-sarcosine-based GlyT1 inhibitor developed by Pfizer (221), was entered into clinical trials and provided positive results for the enhancement of cognitive remediation in schizophrenia (201). However, the first phase II clinical trial (203) on the use of PF-3463275 as an add-on therapy for the treatment of negative symptoms was terminated because of unspecified scientific reasons and safety concerns. The second phase II clinical trial (202) was initiated in 2013, and although it has remained active, to the best of our knowledge, there has been no recruitment efforts to date.

In addition to the abovementioned non-sarcosine-based GlyT1 inhibitors, bitopertin (also known as RG1678 or RO4917838) is an oral, non-competitive GlyT1 inhibitor that was originally developed by Roche as a potential drug candidate for the treatment of negative symptoms of schizophrenia. Preclinical studies revealed that bitopertin modulated schizophrenia-like behaviors in several naïve and pharmacologically challenged animal models (222, 223). The most promising finding of bitopertin was the result of an 8-week randomized, double-blind, proof-of-concept phase II study, in which bitopertin was proven to be safe, with the results showing an inverted U-shaped dose–response efficacy against the predominant negative symptoms of stable schizophrenia patients (207, 208), but no similar effect was observed in the quality of life of these patients (209). Subsequently, in a phase II/III clinical trial, bitopertin monotherapy improved only the positive subscale score of the PANSS (Positive and Negative Syndrome Scale) with respect to acute exacerbation of schizophrenia (205). In a randomized double-blind phase III study following one-year as an adjunctive treatment, bitopertin was found to be generally safe and well-tolerated for the treatment of Japanese patients with schizophrenia, and all three bitopertin-treated groups showed improvements in all the efficacy endpoints for both “negative symptoms” and “suboptimally controlled symptoms” throughout the duration of the study (210). Except for this study, unfortunately, the superior efficacy over placebo of adjunctive bitopertin at any of the doses tested in patients with persistent predominant negative symptoms of schizophrenia could not be proven in several randomized, double-blind, placebo-controlled phase III trials (204, 206, 211, 212). Furthermore, bitopertin did not significantly affect any symptoms, NMDAR-related biomarkers, or MMN frequency at the doses tested in double-blind clinical trials with patients with schizophrenia (157, 213). Accordingly, the negative results and small improvements associated with bitopertin suggest that adjunctive bitopertin treatment might only offer a modest benefit and that bitopertin might not be a broadly effective or optimal therapeutic candidate for the treatment of schizophrenia. Further study will be needed to elucidate the effect of bitopertin in animal models and clinical trials.

Furthermore, BI 425809 was recently developed by Boehringer Ingelheim as a novel, investigational GlyT1 inhibitor to improve cognitive function and memory in patients with schizophrenia and Alzheimer's disease (224–226). A recent randomized double-blind, placebo-controlled phase II study revealed that BI 425809 improved cognitive functions after 12 weeks in patients with schizophrenia (215), suggesting that BI 425809 can provide an effective treatment for cognitive impairment associated with schizophrenia. Currently, another phase II trial of BI 425809 combined with computerized cognitive training for schizophrenic patients is in progress (214, 227). Further large-scale phase III clinical trials will be necessary to replicate these encouraging findings and to confirm the therapeutic potential of BI 425809 for the treatment of cognitive deficits in schizophrenia.

In summary, both sarcosine-based and non-sarcosine-based GlyT1 inhibitors are generally well-tolerated and exhibit a satisfactory safety profile. GlyT1 inhibitors also exert more-promising therapeutic potential than agonists directly targeting the GMS in the improvement of schizophrenic symptoms. However, in consideration of the etiology and pathophysiology of schizophrenia, no evidence has supported a proposal that GlyT1 is overexpressed in the brains of schizophrenic patients. In contrast, a series of negative findings of association studies have revealed that neither glycine transmission nor GlyT1 is implicated in the pathogenesis of schizophrenia (228–230). As described previously, although concentrations of glycine are 10-fold higher than D-serine, D-serine is considered the dominant endogenous coagonist of NMDARs and a modulator of NMDAR-related neurotoxicity (20, 21). Thus, targeting GlyT1 might not be an optimal strategy for modulation of NMDAR functions. Furthermore, functional distinctions between synaptic and extrasynaptic NMDARs in brain physiology, in which synaptic and extrasynaptic NMDARs are gated by D-serine and glycine, respectively, have been reported (22, 231). D-serine and glycine differentially impact NMDAR membrane diffusion and neuroplasticity (21, 22). Given that glycine, but not D-serine, preferentially gates NMDARs located at extrasynaptic sites and that synaptic, but not extrasynaptic, NMDARs are essential for LTP induction, it is plausible that the efficacy and therapeutic effect of GlyT1 inhibitors might be relatively less effective than those of D-serine. Thus, as an alternative to GlyT1 inhibitors, one of the promising approaches for the development of novel therapeutic compounds to treat schizophrenia is based on increased synaptic D-serine levels realized through the indirect modulation of astrocytic D-serine synthesis.

### Indirect Modulation of NMDAR Functions by Targeting DAO

DAO (or DAAO) encodes D-amino acid oxidase which has a flavin adenine dinucleotide (FAD) as the prosthetic group, and DAO catalyzes the oxidative deamination of a wide range of D-amino acids, including D-serine (232–234). The human *DAO* gene is located on chromosome 12q24, and DAO is mainly expressed in the liver, kidney and CNS (235). DAO is abundant in both neurons and glial cells in the cerebral cortex, hippocampus and cerebellum and contributes to normal neuronal functioning (236, 237). DAO has been of interest in psychiatry because its major substrate in the brain is D-serine, which modulates NMDAR functions and contributes to NMDAR hypofunction in schizophrenia. D-serine is synthesized from L-serine by SR and is metabolized by DAO and SR through an α, β-elimination reaction. Among DAO substrates in the brain, D-serine is clearly the most abundant. DAO is believed to play a crucial role in the regulation of cellular D-serine concentrations and release (143). In particular, the three-dimensional structure of human DAO is a stable homodimer and it is highly conserved compared to the microorganism sources (238, 239). Human DAO possesses a low FAD binding function and mainly presents in an inactive apoprotein form (238, 240) because of its specific structure. DAO also exhibits a low substrate affinity and catalytic efficiency for D-serine (234, 241). The inactive apoprotein form of human DAO prevents excessive degradation of D-serine in the brain. The active holeenzyme of human DAO is reconstituted by binding of active-site ligands, such as FAD and the substrate stabilizes flavin binding, and thus pushing the acquisition of catalytic competence (238, 242). Intriguingly, it has been reported that DAO inhibitor (e.g., benzoate) increases the holoenzyme reconstitution of human DAO and stabilizes the flavoprotein (243). In addition, human DAO is mainly colocalized with pyramidal neurons in the prefrontal cortex and hippocampus (236). Enhanced DAO activity is considered a potential cause of reduced D-serine and subsequent impairment to NMDAR functioning in schizophrenia (123, 244).

The glutamate hypothesis of schizophrenia suggests that increased DAO activity leads to decreased D-serine levels, which may subsequently lead to NMDAR hypofunction. Supporting evidence from association studies, DAO expression in schizophrenic patients and behavioral outcomes observed in rodent models have suggested potential therapeutic benefits of DAO inhibitors (DAOIs). Accumulating evidence from genetic studies has indicated that *DAO* and *G72* are putative genes related to schizophrenia (235, 245, 246). Schizophrenic patients with genetic variation in *DAO* and *G72* genes also display negative valence and cognitive deficits (247–250). In complementary findings, a recent GWAS revealed that of 108 schizophrenia-associated loci, none were within the *DAO* or *G72* gene regions (13). Although reports on the association of *DAO* and *G72* with schizophrenia are ambiguous, these genes remain candidates in schizophrenia because of their roles in glutamatergic signaling, which has been associated with schizophrenia in multiple lines of research (157, 166, 246). Both *G72* mRNA and G72 protein (as known as pLG72) are detected in higher levels in brain and blood of schizophrenia patients (251, 252). Intriguingly, DAO-pLG72 complex was reported to modulate intracellular D-serine concentration in human (233, 238), which suggests a novel avenue to design molecules to regulate human DAO activity and thus NMDAR function for future research. In the same vein, the expression and activity of DAO are significantly increased in patients with schizophrenia (28, 236, 244, 253). Intriguingly, it has been reported that chlorpromazine (i.e., a first-generation antipsychotic) and risperidone (i.e., a second-generation antipsychotic) are potentially active substances that inhibit DAO function (254, 255). In addition, inactivation of DAO in rodents produces behavioral and biochemical effects, suggesting potential therapeutic benefits (143). Indeed, increasing levels of D-serine have been observed in rodents after the administration of DAOIs (256–258). Consistently, PCP- or MK-801-induced pre-pulse inhibition deficits and cognitive deficits relevant to schizophrenia were ameliorated after treatment with DAOIs (256, 259, 260). DAOIs increase the levels of D-alanine, which might also be beneficial for increasing NMDAR function (260). Moreover, ddY/DAO(–) mice, which lack active DAO due to a point mutation, exhibited increased cerebellar NMDAR functions (261), enhanced hippocampal LTP, and improved spatial learning in a water maze (262). Other animal studies have indicated that DAO is involved in the mechanism of D-serine nephrotoxicity (263), which is attenuated by DAOIs (264). D-serine combined with DAOI or DAOI alone might be beneficial for enhancing NMDAR functions in schizophrenia.

In agreement with the abovementioned studies, DAOIs are among the most attractive therapeutic targets for improving cognition and reducing negative symptoms in schizophrenia discovered in recent decades. Basically, DAOIs can be divided into two categories: cofactor-competitive and substrate-competitive inhibitors (238, 240, 241, 265–267). Chlorpromazine, the first antipsychotic medication, is a traditional dopamine D2 receptor antagonist but it has been reported that chlorpromazine is also a FAD-competitive DAO inhibitor (243, 255). Compared to the cofactor-competitive inhibitor of DAO, substrate-competitive DAOIs (such as CBIO and benzoate) are frequently used as scaffolds for developing novel drugs. In the late 2000s, a series of structurally similar molecules (such as ASO57278 (256), Merck compound (257), Pfizer compounds (258), and CBIO (268), displayed a potent inhibition of DAO *in vitro* but had limited elevation of D-serine *in vivo*. Especially, it has been reported that acute and chronic administrations of ASO57278 produced inverted U-shaped dose-response curves to reverse PCP-induced PPI deficits (256). And co-administration of CBIO with D-serine also significantly increased D-serine level and attenuated MK-801 induced PPI deficit (259). Thus, these studies imply that DAOIs have beneficial effects in treatment of schizophrenia.

To date, there are at least two potential DAOIs that have been advanced into clinical evaluation, including sodium benzoate and TAK-831. Effects of these two DAOIs on the treatment of schizophrenic symptoms in clinical studies are summarized in Table 6. Sodium benzoate is known as a preservative that is widely used as a food pickling agent. Sodium benzoate is a prototype competitive inhibitor of DAO, and preclinical studies have indicated that it attenuates PCP-induced pre-pulse inhibition deficits as well as D-serine-induced nephrotoxicity (264, 277). The first randomized, double-blind, placebo-controlled trial with chronic schizophrenia patients reported that add-on sodium benzoate relieved positive, negative, and cognitive symptoms as well as improved quality of life (269). Sodium benzoate also showed efficacy and safety for schizophrenic patients who had a poor response to clozapine (270). Moreover, adjunctive sodium benzoate plus sarcosine, but not sarcosine alone, improved the cognitive and global functioning of chronic schizophrenia patients (199). However, a randomized clinical study in Australia indicated that adjunctive use of sodium benzoate had no effect on individuals with early psychosis (271, 272). Two adaptive clinical phase II studies performed to evaluate the safety and efficacy of sodium benzoate in adolescent schizophrenia patients (273) and treatment-resistant schizophrenia patients (274) are currently recruiting. One probable drawback for the development of sodium benzoate as a drug candidate is that it lacks patentability due to its simple chemical structure. More evidence on the therapeutic effect of sodium benzoate, especially in larger-scale clinical trials in schizophernia, is required to prove its effectiveness and applicability. In addition, another highly selective and potent DAOI from Takeda known as TAK-831 is currently being evaluated for schizophrenia in a phase II clinical trial (275, 276, 278). A series of studies of TAK831, including those directed to pharmacokinetics, target occupancy, and D-serine concentrations in the brain, have detected and analyzed a non-linear quantitative multilayer mechanistic model for multilayer biomarker-assisted clinical development with multiple CNS indications (279). Investigations to discover the characteristics and potential development of TAK-831 are needed to determine its efficacy and tolerability in the management of different domains of schizophrenia. In addition to sodium benzoate and TAK-831, there are additional unpublished data on DAOIs for which patent applications have been filed and which have been claimed to have specific therapeutic utility in the treatment of schizophrenia and other neuropsychiatric disorders (280). It is worth further investigating the safety and therapeutic potential of these novel DAOIs for the treatment of unmet medical needs of patients with in schizophrenia in future studies.

**Table 6:** Potential clinical efficacy and benefits related to D-amino acid oxidase inhibitors (DAOIs) on the treatment of schizophrenic symptoms.

**Compound**	**Type**	**Study site**	**Patient**	**Usage**	**Subject number (placebo vs. experiment)**	**Dosage**	**Trial duration (weeks)**	**Clinical outcomes**	**Clinical ratings**	**References**
Sodium benzoate	DB (Parallel) (NCT00960219)	Taiwan	SZ	Add on	23 (27) vs. 24 (25)	1 (g/day)	6	+ (Positive, negative, general symptoms)	PANSS, SANS, CGI, GAF, MCCB, HDRS, QOLS, SAS, AIMS, BARS, UKU	(269)
	DB (Parallel)	Taiwan	SZ	Add on	16 (21) vs. 17 (21) (Placebo vs. sarcosine + Bezoate)	Sarcosine: 2 (g/day) Benzoate: 1 (g/day)	12	+ (Cognitive symptom)	PANSS, CGI, GAF, MCCB, SAS, AIMS, BARS, UKU	(199)
	DB (Parallel) (NCT01390376)	Taiwan	TRS SZ	Add on (Clozapine)	20 vs. 20 vs. 20 (Placebo vs. 1 vs. 2)	1, 2 (g/day)	6	+ (Positive, negative symptoms)	PANSS, SANS, GAF, MCCB, HDRS, QOLS, SAS, AIMS, BARS, UKU	(270)
	DB (Parallel) (ACTRN12615000187549)	Australia	Early psychosis (SZ, SCHF, delusion, bipolar)	Add on	160 (Total)	1 (g/day)	12	Protocol	PANSS, CGI, GAF, HDRS, AQOL, PAQ, PGI	(271)
					40 (50) vs. 39 (50)			–		(272)
	DB (Parallel) (NCT01908192)	US & Taiwan	SZ (Adolescent)	Add on	126 (Total)	1 (g/day)	6	Recruiting	PANSS, SANS, CGI, CGAS, CDRS-R	(273)
	DB (Parallel) (NCT03094429)	US	TRS SZ	Add on (Clozapine)	287 (Total)	1, 2 (g/day)	8	Recruiting	PANSS, CGI, HDRS, PSP, SQLS, C-SSRS, SAS, AIMS, BARS, C-SSRS	(274)
TAK-831	DB (Crossover) (NCT03359785)	US	SZ	Add on	31 (32) (Total)	50, 500 (mg/day)	8 days	Complete	BACS, EBC, ASSR, ERP (MMN)	(275)
	DB (Parallel) (NCT03382639)	Worldwide	SZ	Add on	307 (315) (Total)	50, 125, 500 (mg/day)	12	Complete	PANSS, BNSS, BACS, CGI, SCoRS	(276)

*+, Positive clinical results; −, Negative clinical results; AIMS, Abnormal Involuntary Movements Scale; AQOL, Assessment of Quality of Life; ASSR, Auditory Steady State Response; BACS, Brief Assessment of Cognition in Schizophrenia; BARS, Barnes Akathisia Rating Scale; BNSS, Brief Negative Symptom Scale; CDRS-R, Children's Depression Rating Scale-Revised; CGAS, Children's Global Assessment Scale; CGI, Clinical Global Impression; C-SSRS, Columbia-Suicide Severity Rating Scale; DB, double-blind; EBC, Eye Blink Conditioning; ERP, Event Related Potential; GAF, Global Assessment of Function; HDRS, Hamilton Depression Rating Scale; MCCB, MATRICS consensus cognitive battery; MMN, Mismatch negativity; PANSS, Positive and Negative Syndrome Scale; PAQ, Physical Activity Questionnaire; PGI, Patient Global Impression; PSP, Personal and Social Performance scale; QOLS, Quality of Life Scale; SANS, Scale for the Assessment of Negative Symptoms; SAS, Simpson Angus Scale for Assessment of Extrapyramidal Side Effects; SCoRS, Schizophrenia Cognition Rating Scale; SQLS, Schizophrenia Quality of Life Scale; SZ, schizophrenia; SCHF, Schizophreniform disorder; TRS, treatment-resistant; UKU, Udvalg for Kliniske Undersogelser Side Effects Rating Scale*.

## Conclusion

**Figure 2:**
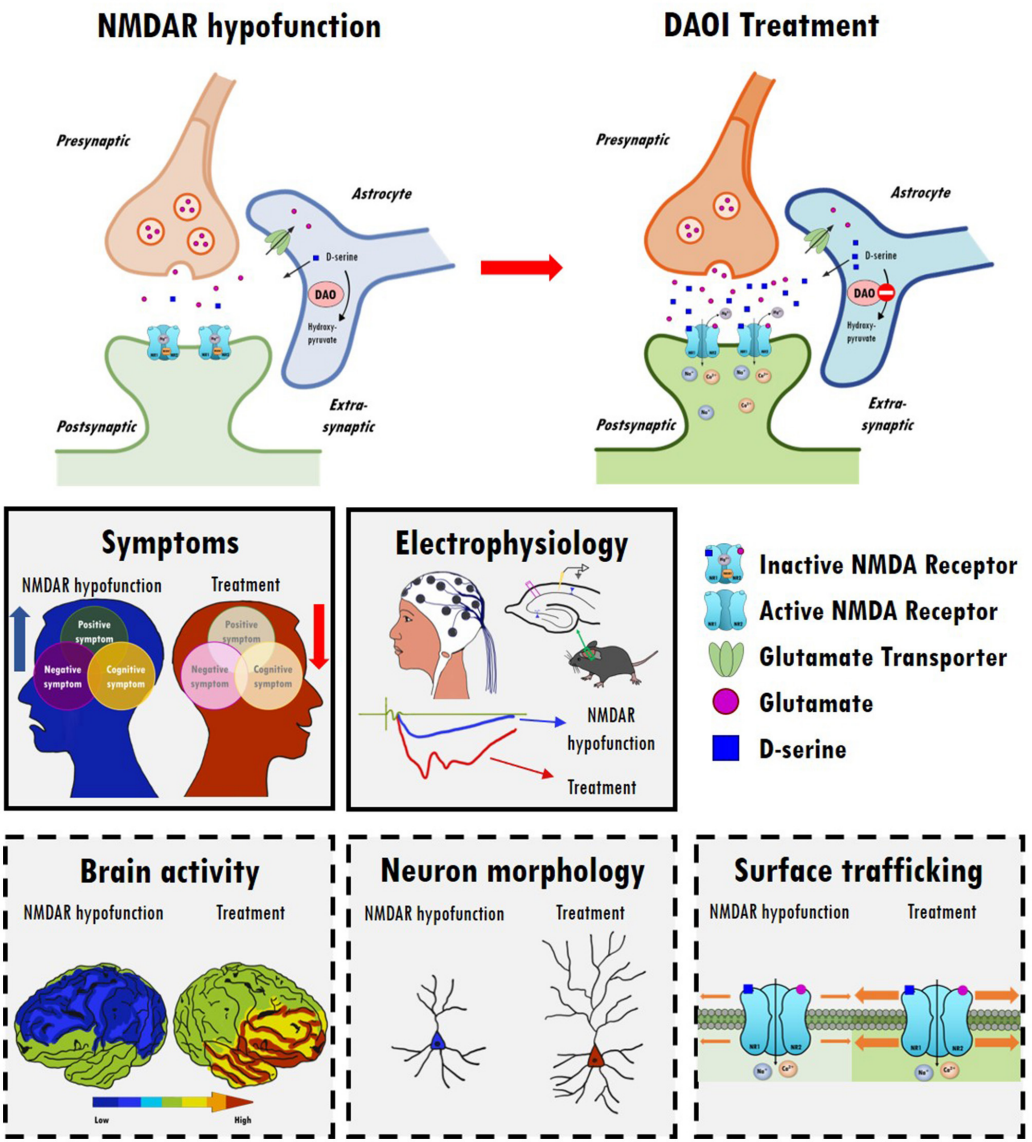
The therapeutic effects and possible underlying mechanisms of D-amino acid oxidase inhibitors (DAOIs) in the treatment of schizophrenia. Top panel: Indirect modulation of NMDAR functions by DAOIs restores NMDAR hypofunction. Inhibition of DAO results in increased synaptic levels of D-serine. Middle panels: DAOIs significantly alleviate positive, negative, and cognitive symptoms in patients with schizophrenia and moderate schizophrenia-like behavioral deficits in animal models. DAOIs enhance NMDAR functions and hippocampal LTP in animal studies. Bottom panels: Possible mechanism of action of DAOIs. The effects of DAOIs on brain activity, neuromorphology, and cell surface trafficking of NMDARs, which contribute to the amelioration of NMDAR hypofunction and restoration of mental functions, are worthy of further investigation.

Data from clinical, genetic, postmortem, and animal studies strongly implicate NMDARs as central hubs for many pathophysiological processes in the brains of schizophrenic patients. Notably, several NMDAR-enhancing agents, particularly those directed to the GMS of NMDARs, result in the significant alleviation of schizophrenia-like behavioral deficits and cognitive dysfunctions in animal models as well as in patients with schizophrenia. There is great interest in identifying potential drug candidates targeting the GMS of NMDARs and to evaluate their therapeutic effectiveness in attenuating the negative and cognitive symptoms of schizophrenia with minimal adverse effects. Modulation of NMDAR functions through the GMS has been proposed as a possible therapeutic approach to drug development, and either direct or indirect activation of GMS results in differential benefits and adverse effects in the treatment of schizophrenia. A summary of the relevant animal study data, as well as those from clinical trials, examining the therapeutic effects and experimental outcomes of direct and indirect GMS modulators is provided in this article. Overall, current findings suggest that indirectly targeting GMS appears to be more beneficial and results in fewer adverse effects than directly targeting GMS to modulate NMDAR functions. In particular, compared with GlyT1 inhibitors, one of the promising approaches to the development of novel therapeutic compounds for treating schizophrenia is to indirectly increase synaptic D-serine levels by targeting DAO. As illustrated in Figure 2, inhibition of DAO via DAOIs not only results in increased synaptic D-serine levels but also the regulation of NMDAR-evoked electrophysiological activity, which contributes to the amelioration of NMDAR hypofunction and restoration of mental functions. There is great interest in further investigating the effects of DAOIs on brain activity, neuromorphology, and cell surface trafficking of NMDARs, which contribute to the amelioration of NMDAR hypofunction and untreated symptoms of schizophrenia. Thus, GMS modulators, especially GlyT1 inhibitors and DAOIs, may open new avenues to the treatment of unmet medical needs in patients with schizophrenia, which is worthy of further investigation. For the development of new antipsychotic drugs, the establishment of safety profiles of these potential compounds will be beneficial and informative, possibly leading to the elucidation of their precise mechanisms of action and the evaluation of their therapeutic effects in both animal models and clinical studies. Notably, however, this review presents an oversimplified summary of the treatment alternatives for an extremely complex psychiatric disorder. Indeed, human diseases are far more complex and only some aspects of human diseases can be partially modeled in animal models. Clinical trials are essential and irreplaceable in drug development. In complementary to human studies, preclinical animal studies are highly valuable and indispensable to the understanding of the underlying mechanism and for the development of new drugs. And we simply focus on discussing the importance of NMDAR functions on excitatory rather than inhibitory neurons in this review article. The role of inhibitory neurons and the impact of NMDAR hypofunction on GABAergic neurons in the pathophysiology of schizophrenia are worth further investigating (281, 282). Because the etiology of schizophrenia remains unclear, disturbances to the GABAergic, cholinergic, and dopaminergic neurotransmitter systems (283, 284), as well as disruptions to astrocyte function (164), are also worthy of further investigation.

## Author Contributions

J-CP and D-ZL are the key persons to wrote and prepare this review article. S-SG collected data and prepared tables. C-YC wrote some paragraphs of this article. W-SL organized, wrote, and modified the whole article. All authors contributed to the article and approved the submitted version.

## Funding

This research was supported by Grant Nos. 109-2918-I-002-008, 109-2926-I-002-509, 109-2410-H-002-087-MY3, and 110-2410-H-002-235-MY3 from the Ministry of Science and Technology in Taiwan to W-SL and by grant support from National Taiwan University and National Taiwan University Hospital.

## Conflict of Interest

The authors declare that the research was conducted in the absence of any commercial or financial relationships that could be construed as a potential conflict of interest.

## Publisher's Note

All claims expressed in this article are solely those of the authors and do not necessarily represent those of their affiliated organizations, or those of the publisher, the editors and the reviewers. Any product that may be evaluated in this article, or claim that may be made by its manufacturer, is not guaranteed or endorsed by the publisher.
